# 11*H*-Indeno­[1,2-*b*]quinoxalin-11-one

**DOI:** 10.1107/S1600536810019252

**Published:** 2010-05-29

**Authors:** Raza Murad Ghalib, Rokiah Hashim, Othman Sulaiman, Madhukar Hemamalini, Hoong-Kun Fun

**Affiliations:** a School of Industrial Technology, Universiti Sains Malaysia, 11800 USM, Penang, Malaysia; b X-ray Crystallography Unit, School of Physics, Universiti Sains Malaysia, 11800 USM, Penang, Malaysia

## Abstract

In the title compound, C_15_H_8_N_2_O, the fused ring system is approximately planar, with a maximum deviation of 0.039 (1) Å. In the crystal, weak inter­molecular C—H⋯O inter­actions help to establish the packing.

## Related literature

For applications of and background to indeno­quinoxaline, see: Gazit *et al.* (1996 ▶); Sehlstedt *et al.* (1998 ▶). For a related structure, see: Leslie *et al.* (1993 ▶). For the stability of the temperature controller used in the data collection, see: Cosier & Glazer (1986 ▶).
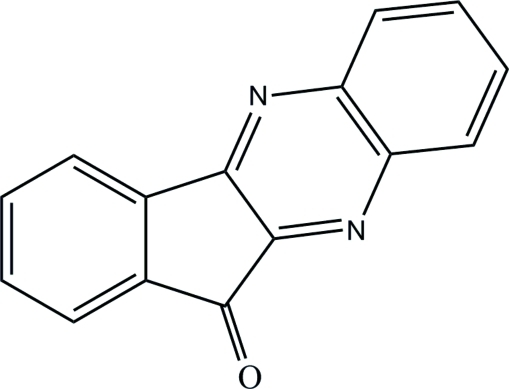


## Experimental

### 

#### Crystal data

C_15_H_8_N_2_O
*M* *_r_* = 232.23
Orthorhombic, 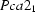

*a* = 23.688 (3) Å
*b* = 3.7862 (5) Å
*c* = 11.5730 (16) Å
*V* = 1038.0 (2) Å^3^
*Z* = 4
Mo *K*α radiation
μ = 0.10 mm^−1^
*T* = 100 K
0.65 × 0.17 × 0.09 mm

#### Data collection

Bruker APEXII DUO CCD diffractometer
Absorption correction: multi-scan (*SADABS*; Bruker, 2009 ▶) *T*_min_ = 0.940, *T*_max_ = 0.991
8012 measured reflections
2004 independent reflections
1879 reflections with *I* > 2σ(*I*)
*R*_int_ = 0.031

#### Refinement

*R*[*F* ^2^ > 2σ(*F* ^2^)] = 0.036
*wR*(*F* ^2^) = 0.096
*S* = 1.05
2004 reflections
195 parameters
1 restraint
H atoms treated by a mixture of independent and constrained refinement
Δρ_max_ = 0.35 e Å^−3^
Δρ_min_ = −0.23 e Å^−3^

### 

Data collection: *APEX2* (Bruker, 2009 ▶); cell refinement: *SAINT* (Bruker, 2009 ▶); data reduction: *SAINT*; program(s) used to solve structure: *SHELXTL* (Sheldrick, 2008 ▶); program(s) used to refine structure: *SHELXTL*; molecular graphics: *SHELXTL*; software used to prepare material for publication: *SHELXTL* and *PLATON* (Spek, 2009 ▶).

## Supplementary Material

Crystal structure: contains datablocks global, I. DOI: 10.1107/S1600536810019252/hb5459sup1.cif

Structure factors: contains datablocks I. DOI: 10.1107/S1600536810019252/hb5459Isup2.hkl

Additional supplementary materials:  crystallographic information; 3D view; checkCIF report

## Figures and Tables

**Table 1:** Hydrogen-bond geometry (Å, °)

*D*—H⋯*A*	*D*—H	H⋯*A*	*D*⋯*A*	*D*—H⋯*A*
C3—H3*A*⋯O1^i^	0.96 (2)	2.55 (2)	3.401 (2)	148.3 (19)
C9—H9*A*⋯O1^ii^	0.97 (3)	2.49 (3)	3.2458 (18)	134.0 (18)

Symmetry codes: (i) 
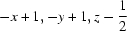
; (ii) 
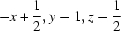
.

**Fig. 1.**
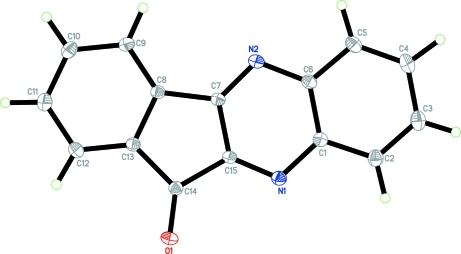
The asymmetric unit of (I), showing 50% probability displacement ellipsoids.

**Fig. 2.**
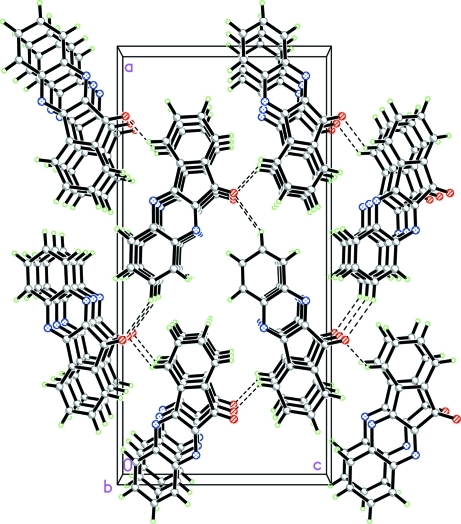
The crystal packing of (I), showing the hydrogen-bond (dashed lines) network.

## supplementary crystallographic information

### Comment

Indenoquinoxaline derivatives are important classes of nitrogen containing heterocycles and they constitute useful intermediates in organic synthesis (Gazit *et al.*, 1996).  They have been reported for their applications in dyes and have also been used as building blocks for the synthesis of organic semiconductors. More interestingly, research has revealed that these compounds exhibit diverse medicinal functions such as antimetabolism and antitubercular properties (Sehlstedt *et al.*, 1998). In view of the biological importance of indenoquinoxalines, we report here the crystal structure of the title compound, (I).

The molecule of indeno[1,2-*b*]quinoxalin-11-one (Fig. 1) is approximately planar with maximum deviation of 0.039 (1) Å for atom C14. It contains three ring systems, viz., indene (C7–C15), pyrazine (N1/N2/C6–C7/C1/C15) and benzene (C1–C6). The C–N bond distances and C—N—C angles are C15—N1 = 1.3070 (17) Å, C1—N1 = 1.3793 (18) Å, C7—N2 = 1.3142 (17) Å, C6—N2 = 1.3800 (17) Å, C15—N1—C1 = 113.99 (12)° and C7—N2—C6 = 114.00 (12)°. These values agree with those reported in the related structure of 11*H*-indeno[1,2-*b*]quinoxalin-11-ones (Leslie *et al.*, 1993). The pyrazine (N1/N2/C6–C7/C1/C15) ring makes dihedral angles of 0.48 (5)° and 1.34 (6)° with the indene (C7–C15) ring and the benzene (C1–C6) ring, respectively. The dihedral angle between the indene (C7–C15) ring and benzene (C1–C6) ring is 0.88 (6)°.

In the crystal structure, molecules are linked by weak intermolecular C3—H3A···O1 and C9—H9A···O1 hydrogen bonds  (Table 1) interactions which help to stabilize the crystal structure.

### Experimental

The title compound, has been synthesized by two routes: a mixture of ninhydrin (1.78 g) and *o*-phenylenediamine (1.08 g) in molar ratio 1:1 were [a] stirred in distilled water for 15 minutes and [b] refluxed in THF for 1 hour in presence of HCl. Both these mixtures were separately dried on rota-vapor at low pressure and then crystallized from chloroform-n-hexane (1:1) to give yellowish needles of (I).

### Refinement

Anomalous dispersion was negligible and 1465 Friedel pairs were merged for the final refinement. All the H atoms were located in a difference Fourier map and allowed to refine freely [C—H = 0.96 (2)–1.00 (2) Å].

### Crystal data

**Table:**

C_15_H_8_N_2_O	*F*(000) = 480
*M**_r_* = 232.23	*D*_x_ = 1.486 Mg m^−^^3^
Orthorhombic, *P**c**a*2_1_	Mo *K*α radiation, λ = 0.71073 Å
Hall symbol: P 2c -2ac	Cell parameters from 3067 reflections
*a* = 23.688 (3) Å	θ = 3.4–32.7°
*b* = 3.7862 (5) Å	µ = 0.10 mm^−^^1^
*c* = 11.5730 (16) Å	*T* = 100 K
*V* = 1038.0 (2) Å^3^	Needle, yellow
*Z* = 4	0.65 × 0.17 × 0.09 mm

### Data collection

**Table:**

Bruker APEXII DUO CCD diffractometer	2004 independent reflections
Radiation source: fine-focus sealed tube	1879 reflections with *I* > 2σ(*I*)
graphite	*R*_int_ = 0.031
φ and ω scans	θ_max_ = 32.9°, θ_min_ = 3.4°
Absorption correction: multi-scan (*SADABS*; Bruker, 2009)	*h* = −34→35
*T*_min_ = 0.940, *T*_max_ = 0.991	*k* = −5→5
8012 measured reflections	*l* = −17→14

### Refinement

**Table:**

Refinement on *F*^2^	Primary atom site location: structure-invariant direct methods
Least-squares matrix: full	Secondary atom site location: difference Fourier map
*R*[*F*^2^ > 2σ(*F*^2^)] = 0.036	Hydrogen site location: inferred from neighbouring sites
*wR*(*F*^2^) = 0.096	H atoms treated by a mixture of independent and constrained refinement
*S* = 1.05	*w* = 1/[σ^2^(*F*_o_^2^) + (0.0723*P*)^2^] where *P* = (*F*_o_^2^ + 2*F*_c_^2^)/3
2004 reflections	(Δ/σ)_max_ < 0.001
195 parameters	Δρ_max_ = 0.35 e Å^−^^3^
1 restraint	Δρ_min_ = −0.23 e Å^−^^3^

### Special details

**Table:**

Experimental. The crystal was placed in the cold stream of an Oxford Cryosystems Cobra open-flow nitrogen cryostat (Cosier & Glazer, 1986) operating at 100.0 (1) K.
Geometry. All s.u.'s (except the s.u. in the dihedral angle between two l.s. planes) are estimated using the full covariance matrix. The cell s.u.'s are taken into account individually in the estimation of s.u.'s in distances, angles and torsion angles; correlations between s.u.'s in cell parameters are only used when they are defined by crystal symmetry. An approximate (isotropic) treatment of cell s.u.'s is used for estimating s.u.'s involving l.s. planes.
Refinement. Refinement of F^2^ against ALL reflections. The weighted R-factor wR and goodness of fit S are based on F^2^, conventional R-factors R are based on F, with F set to zero for negative F^2^. The threshold expression of F^2^ > 2σ(F^2^) is used only for calculating R-factors(gt) etc. and is not relevant to the choice of reflections for refinement. R-factors based on F^2^ are statistically about twice as large as those based on F, and R- factors based on ALL data will be even larger.

### Fractional atomic coordinates and isotropic or equivalent isotropic displacement parameters (Å^2^)

**Table:**

	*x*	*y*	*z*	*U*_iso_*/*U*_eq_	
O1	0.33655 (5)	0.7879 (3)	1.04339 (10)	0.0172 (2)	
N1	0.42154 (5)	0.4986 (3)	0.87194 (11)	0.0133 (2)	
N2	0.35873 (5)	0.1857 (3)	0.68337 (10)	0.0133 (2)	
C1	0.44805 (5)	0.3540 (4)	0.77697 (12)	0.0124 (2)	
C2	0.50791 (6)	0.3630 (4)	0.77183 (14)	0.0159 (2)	
C3	0.53554 (6)	0.2296 (4)	0.67669 (15)	0.0181 (3)	
C4	0.50483 (6)	0.0792 (4)	0.58433 (14)	0.0178 (3)	
C5	0.44661 (6)	0.0648 (4)	0.58754 (13)	0.0161 (2)	
C6	0.41693 (6)	0.2018 (3)	0.68361 (12)	0.0129 (2)	
C7	0.33505 (5)	0.3278 (3)	0.77494 (12)	0.0113 (2)	
C8	0.27433 (5)	0.3583 (3)	0.80211 (12)	0.0117 (2)	
C9	0.22743 (6)	0.2529 (4)	0.73926 (13)	0.0141 (2)	
C10	0.17415 (6)	0.3143 (4)	0.78798 (14)	0.0162 (3)	
C11	0.16835 (6)	0.4757 (4)	0.89631 (14)	0.0166 (3)	
C12	0.21568 (6)	0.5858 (4)	0.95898 (14)	0.0145 (2)	
C13	0.26863 (5)	0.5257 (3)	0.91037 (13)	0.0120 (2)	
C14	0.32527 (5)	0.6219 (3)	0.95612 (12)	0.0122 (2)	
C15	0.36646 (6)	0.4822 (3)	0.86782 (12)	0.0116 (2)	
H2A	0.5309 (9)	0.475 (6)	0.834 (3)	0.024 (6)*	
H3A	0.5758 (10)	0.236 (6)	0.671 (2)	0.022 (5)*	
H4A	0.5277 (11)	−0.002 (7)	0.519 (3)	0.033 (7)*	
H5A	0.4213 (10)	−0.031 (6)	0.527 (3)	0.025 (6)*	
H9A	0.2305 (9)	0.133 (6)	0.665 (2)	0.024 (6)*	
H10A	0.1405 (11)	0.235 (6)	0.746 (2)	0.024 (6)*	
H11A	0.1301 (12)	0.493 (7)	0.927 (2)	0.035 (7)*	
H12A	0.2086 (14)	0.697 (8)	1.033 (3)	0.048 (8)*	

### Atomic displacement parameters (Å^2^)

**Table:**

	*U*^11^	*U*^22^	*U*^33^	*U*^12^	*U*^13^	*U*^23^
O1	0.0188 (4)	0.0209 (5)	0.0120 (5)	−0.0003 (4)	−0.0011 (4)	−0.0053 (4)
N1	0.0141 (5)	0.0137 (5)	0.0121 (5)	0.0003 (4)	−0.0006 (4)	−0.0002 (4)
N2	0.0165 (5)	0.0125 (5)	0.0108 (5)	0.0005 (4)	−0.0005 (4)	−0.0006 (4)
C1	0.0136 (5)	0.0118 (5)	0.0119 (5)	0.0001 (4)	−0.0002 (4)	0.0004 (4)
C2	0.0154 (5)	0.0150 (5)	0.0173 (6)	−0.0003 (5)	0.0013 (5)	0.0007 (5)
C3	0.0160 (5)	0.0164 (6)	0.0217 (7)	0.0021 (5)	0.0039 (5)	0.0026 (5)
C4	0.0197 (6)	0.0157 (6)	0.0181 (6)	0.0021 (5)	0.0063 (5)	0.0014 (5)
C5	0.0195 (6)	0.0137 (6)	0.0150 (6)	0.0015 (5)	0.0026 (5)	−0.0010 (4)
C6	0.0156 (5)	0.0117 (5)	0.0113 (6)	0.0004 (4)	0.0006 (5)	0.0002 (4)
C7	0.0132 (5)	0.0103 (5)	0.0103 (5)	0.0007 (4)	−0.0007 (4)	0.0002 (4)
C8	0.0133 (5)	0.0114 (5)	0.0106 (5)	0.0006 (4)	−0.0011 (4)	0.0001 (4)
C9	0.0149 (5)	0.0135 (5)	0.0139 (6)	−0.0006 (4)	−0.0027 (4)	−0.0007 (4)
C10	0.0138 (5)	0.0144 (6)	0.0204 (7)	−0.0012 (4)	−0.0025 (5)	0.0012 (5)
C11	0.0146 (5)	0.0161 (6)	0.0193 (7)	0.0004 (4)	0.0012 (5)	0.0012 (5)
C12	0.0159 (5)	0.0142 (5)	0.0135 (6)	0.0005 (4)	0.0021 (5)	0.0000 (5)
C13	0.0132 (5)	0.0115 (5)	0.0111 (5)	−0.0001 (4)	−0.0004 (4)	0.0002 (4)
C14	0.0128 (5)	0.0128 (5)	0.0112 (5)	0.0003 (4)	−0.0006 (4)	0.0002 (4)
C15	0.0135 (5)	0.0121 (5)	0.0092 (5)	0.0001 (4)	−0.0007 (4)	−0.0005 (4)

### Geometric parameters (Å, °)

**Table:**

O1—C14	1.2193 (18)	C7—C15	1.4321 (19)
N1—C15	1.3070 (17)	C7—C8	1.4768 (17)
N1—C1	1.3793 (18)	C8—C9	1.3865 (18)
N2—C7	1.3142 (17)	C8—C13	1.4106 (19)
N2—C6	1.3800 (17)	C9—C10	1.402 (2)
C1—C2	1.4197 (17)	C9—H9A	0.97 (3)
C1—C6	1.4292 (18)	C10—C11	1.401 (2)
C2—C3	1.377 (2)	C10—H10A	0.98 (3)
C2—H2A	0.99 (3)	C11—C12	1.399 (2)
C3—C4	1.413 (2)	C11—H11A	0.98 (3)
C3—H3A	0.96 (2)	C12—C13	1.3935 (18)
C4—C5	1.381 (2)	C12—H12A	0.97 (3)
C4—H4A	0.98 (3)	C13—C14	1.4876 (18)
C5—C6	1.4140 (19)	C14—C15	1.509 (2)
C5—H5A	1.00 (3)		
			
C15—N1—C1	113.99 (12)	C9—C8—C7	130.26 (13)
C7—N2—C6	114.00 (12)	C13—C8—C7	108.50 (11)
N1—C1—C2	118.59 (13)	C8—C9—C10	117.57 (14)
N1—C1—C6	121.85 (12)	C8—C9—H9A	122.5 (13)
C2—C1—C6	119.55 (13)	C10—C9—H9A	119.9 (13)
C3—C2—C1	119.96 (14)	C11—C10—C9	121.35 (13)
C3—C2—H2A	118.1 (14)	C11—C10—H10A	119.6 (15)
C1—C2—H2A	121.9 (14)	C9—C10—H10A	119.0 (15)
C2—C3—C4	120.54 (13)	C12—C11—C10	121.02 (13)
C2—C3—H3A	121.2 (16)	C12—C11—H11A	122.4 (16)
C4—C3—H3A	118.3 (16)	C10—C11—H11A	116.5 (16)
C5—C4—C3	120.66 (13)	C13—C12—C11	117.61 (14)
C5—C4—H4A	124.1 (17)	C13—C12—H12A	126 (2)
C3—C4—H4A	115.2 (17)	C11—C12—H12A	117 (2)
C4—C5—C6	120.21 (14)	C12—C13—C8	121.20 (13)
C4—C5—H5A	126.7 (15)	C12—C13—C14	128.92 (14)
C6—C5—H5A	113.1 (15)	C8—C13—C14	109.87 (11)
N2—C6—C5	118.62 (13)	O1—C14—C13	128.22 (13)
N2—C6—C1	122.31 (12)	O1—C14—C15	126.88 (12)
C5—C6—C1	119.07 (12)	C13—C14—C15	104.86 (11)
N2—C7—C15	123.41 (13)	N1—C15—C7	124.43 (13)
N2—C7—C8	128.27 (12)	N1—C15—C14	127.18 (12)
C15—C7—C8	108.32 (12)	C7—C15—C14	108.39 (12)
C9—C8—C13	121.23 (12)		
			
C15—N1—C1—C2	179.01 (12)	C8—C9—C10—C11	−0.1 (2)
C15—N1—C1—C6	0.06 (18)	C9—C10—C11—C12	0.9 (2)
N1—C1—C2—C3	−178.21 (13)	C10—C11—C12—C13	−0.7 (2)
C6—C1—C2—C3	0.8 (2)	C11—C12—C13—C8	−0.3 (2)
C1—C2—C3—C4	−0.8 (2)	C11—C12—C13—C14	178.39 (13)
C2—C3—C4—C5	0.3 (2)	C9—C8—C13—C12	1.12 (19)
C3—C4—C5—C6	0.2 (2)	C7—C8—C13—C12	−179.14 (12)
C7—N2—C6—C5	−178.35 (12)	C9—C8—C13—C14	−177.81 (12)
C7—N2—C6—C1	1.21 (18)	C7—C8—C13—C14	1.93 (14)
C4—C5—C6—N2	179.42 (13)	C12—C13—C14—O1	−3.7 (2)
C4—C5—C6—C1	−0.1 (2)	C8—C13—C14—O1	175.12 (13)
N1—C1—C6—N2	−0.9 (2)	C12—C13—C14—C15	178.69 (14)
C2—C1—C6—N2	−179.87 (13)	C8—C13—C14—C15	−2.50 (14)
N1—C1—C6—C5	178.63 (12)	C1—N1—C15—C7	0.42 (19)
C2—C1—C6—C5	−0.32 (19)	C1—N1—C15—C14	−178.69 (12)
C6—N2—C7—C15	−0.74 (18)	N2—C7—C15—N1	−0.1 (2)
C6—N2—C7—C8	179.53 (12)	C8—C7—C15—N1	179.70 (12)
N2—C7—C8—C9	−1.1 (2)	N2—C7—C15—C14	179.18 (12)
C15—C7—C8—C9	179.17 (14)	C8—C7—C15—C14	−1.05 (14)
N2—C7—C8—C13	179.22 (13)	O1—C14—C15—N1	3.7 (2)
C15—C7—C8—C13	−0.53 (14)	C13—C14—C15—N1	−178.64 (12)
C13—C8—C9—C10	−0.89 (19)	O1—C14—C15—C7	−175.53 (13)
C7—C8—C9—C10	179.44 (13)	C13—C14—C15—C7	2.13 (14)

### Hydrogen-bond geometry (Å, °)

**Table:**

*D*—H···*A*	*D*—H	H···*A*	*D*···*A*	*D*—H···*A*
C3—H3A···O1^i^	0.96 (2)	2.55 (2)	3.401 (2)	148.3 (19)
C9—H9A···O1^ii^	0.97 (3)	2.49 (3)	3.2458 (18)	134.0 (18)

Symmetry codes: (i) −*x*+1, −*y*+1, *z*−1/2; (ii) −*x*+1/2, *y*−1, *z*−1/2.
